# Does variability in automated perfusion software outputs for acute ischemic stroke matter? Reanalysis of EXTEND perfusion imaging

**DOI:** 10.1111/cns.13756

**Published:** 2021-11-16

**Authors:** Andrew Bivard, Leonid Churilov, Henry Ma, Christopher Levi, Bruce Campbell, Nawaf Yassi, Atte Meretoja, Henry Zhao, Gagan Sharma, Chushuang Chen, Stephen Davis, Geoffrey Donnan, Bernard Yan, Mark Parsons, the EXTEND investigators

**Affiliations:** ^1^ Department of Medicine and Neurology Melbourne Brain Centre at the Royal Melbourne Hospital University of Melbourne Parkville VIC Australia; ^2^ Department of Medicine School of Clinical Science Monash University Clayton VIC Australia; ^3^ Department of Neurology John Hunter Hospital Hunter Medical Research Institute University of Newcastle Newcastle NSW Australia; ^4^ Department of Neurology Helsinki University Hospital Helsinki Finland; ^5^ School of Medicine and Public Health University of Newcastle Newcastle NSW Australia; ^6^ Department of Neurology Liverpool Hospital Ingham Institute of Applied Medical Research University of New South Wales South Western Sydney Clinical School Liverpool NSW Australia

**Keywords:** CT perfusion, ischemic stroke, target mismatch, thrombolysis

## Abstract

**Aims:**

We reprocessed the Extending the time for Thrombolysis in Emergency Neurological Deficits (EXTEND) perfusion imaging with a different automated software with the aim of comparing mismatch eligibility and outcomes.

**Methods:**

EXTEND baseline perfusion imaging data were reprocessed using autoMIStar software to identify patients who were eligible based on the same target mismatch criteria as per the original trial.

**Results:**

From the 225 patients fulfilling RAPID‐based mismatch criteria randomized in the EXTEND study, 196 (87%) patients met the revised mismatch criteria. Most common reasons for not meeting revised criteria were core >70 ml (*n* = 9), and no perfusion lesion/lack of penumbral tissue (*n* = 20). The revised perfusion lesion volumes were significantly smaller compared to the original RAPID volumes (median 68 ml IQR 34–102 ml vs. 42 ml 16–92 ml, *p* = 0.036). Of the patients who met the revised mismatch criteria, 40% receiving alteplase had modified Rankin Scale (mRS) 0–1 at 3‐month compared to 28% with placebo (Adjusted Odds Ratio (OR) = 2.23, CI 1.08–4.58, *p* = 0.028). In contrast, in the original mismatch cohort, 35% receiving alteplase had mRS 0–1 at 3‐month compared to 30% with placebo (adjusted OR = 1.88, *p* = 0.056).

**Conclusions:**

These data reinforce the benefit of alteplase in the later time window, and suggest that differences in automated perfusion imaging software outputs may be clinically relevant.

## Graphical abstract

From the 225 patients fulfilling RAPID‐based mismatch criteria randomized in EXTEND, 196 patients met revised mismatch criteria. Of the patients who met revised mismatch criteria, the alteplase group has a higher chance of achieving an excellent outcome than the placebo. Our study reinforces the benefit of alteplase in the later time window in patients selected with perfusion imaging and indicates that differences in automated perfusion imaging software outputs may be clinically relevant.

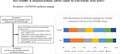

## 1 INTRODUCTION

The use of automated perfusion imaging to identify treatment responders was central in the recent DAWN,^1^ DEFUSE 3,^2^ and EXTEND^3^ clinical trials, which have successfully expanded the treatment time windows for thrombectomy and thrombolysis. The most recent of these, EXTEND, used an American FDA‐approved software RAPID, with Tmax and cerebral blood flow (CBF) defining penumbra and core, respectively, for computer tomographic perfusion (CTP). The pre‐specified primary outcome analysis of EXTEND, using a covariate‐adjusted modified Poisson regression, revealed a statistically significant treatment effect (adjusted Risk Ratio [aRR] = 1.44, CI 1.01–2.06, *p* = 0.04), although on commonly used logistic regression analysis, the odds of achieving excellent functional outcome did not reach significance.^4^

An important and often underexamined element of imaging selected trials is that there is variation between vendor software, and in the case of perfusion imaging software the estimation of the ischemic core and penumbral lesion volumes are likely to be quite different. Each software uses differing algorithms and post‐processing “smoothing,” although it is uncertain if these differences are clinically significant.^4^, ^5^, ^6^ One particular difference between perfusion algorithms is how the “delay” in contrast arrival to ischemic brain tissue is dealt with.^7^ In acute ischemia with a vessel occlusion, there will be significant delay and dispersion in contrast arriving to the ischemic region, particularly if contrast travels retrogradely via leptomeningeal collaterals.^8^ In addition to the delay, there is also “dispersion” of the contrast bolus due to it traveling via various collateral routes. If Delay and Dispersion are not corrected for, this can underestimate CBF, and potentially lead to overestimation of core volume. Use of Tmax to assess penumbra (as this measure does not correct for Delay and Dispersion) may also potentially lead to overestimation of the penumbra.^9^ These may not be clinically significant issues, when there is very large perfusion‐core mismatch. However, where the volume of tissue at risk is smaller and close to the threshold allowed for inclusion in clinical trials (>10–15 ml), differences in software calculation of “penumbra” may be crucial for patient selection. This problem of penumbral overestimation due to delay and dispersion may be particularly relevant in patients with intra or extra‐cranial atherosclerotic disease with stenosis, or impaired cardiac output (including atrial fibrillation), or leukoaraiosis, which is clinically not uncommon.^10^

The aims of this study were, firstly, to determine if there were differences between automated perfusion imaging software (autoMIStar another American FDA‐approved automated, commercially available software) in terms of patients fulfilling volumetric target mismatch eligibility for the EXTEND trial. Secondly, we aimed to identify whether the outcomes for alteplase versus placebo patients who had target mismatch based on autoMIStar differed to those classified as having target mismatch by RAPID.

## 2 MATERIAL AND METHODS

EXTEND was a phase III, investigator driven, multi‐center, randomized controlled study of patients who presented with acute ischemic stroke within 4.5–9 h from symptom onset (ClinicalTrials.gov numbers, NCT00887328 and NCT01580839). The study protocol, patient inclusion/exclusion criteria and original results of the EXTEND trial have been published previously.^3^, ^11^ Patients had to fulfill target mismatch criteria, which were assessed by either magnetic resonance perfusion (MR‐PWI) or CTP imaging using automated commercially available software (RAPID, version 4.7, IschemaView). The target mismatch criteria were an ischemic core volume of <70 ml, penumbral volume >10 ml and a mismatch (perfusion lesion/core) ratio of >1.2. The ischemic core was defined on CTP by rCBF < 30%. The perfusion lesion was defined on perfusion MRI or CTP as the Tmax more than 6 s delay (Tmax > 6 s). Penumbral tissue was defined as tissue within the Tmax > 6 s lesion volume that was not within the ischemic core lesion. Patients were randomized to either alteplase (0.9 mg/kg) or placebo. The primary outcome of EXTEND was the proportion of patients achieving an excellent functional outcome (modified Rankin Scale [mRS] of 0–1 at 90 days), adjusted for age and pre‐treatment National Institutes of Health Stroke Scale (NIHSS).

The study was approved by institutional ethics committee at each participating sites. Written consent was obtained. While the study allowed enrollment with baseline MRI, no patients in this analysis had suitable perfusion imaging on MRI and no patients were enrolled in the EXTEND trial with baseline MRI. The acquisition parameters of the baseline imaging enrollment are included in the EXTEND paper^3^ and required a perfusion sequence of >45 s.

### 2.1 Study measurements

For the current study, individual patient imaging was analyzed, without manual interference, by automated commercially available software (autoMIStar) and was blind to clinical data, follow‐up imaging data, and treatment allocation. All perfusion imaging was processed with a singular value deconvolution algorithm with delay and dispersion correction to generate maps of CBF, cerebral blood volume, mean transit time, and delay time (DT). As previously validated, autoMIStar defines the perfusion lesion as tissue with a DT of >3 s, and the ischemic core as tissue within the perfusion lesion (DT >3 s) but with a CBF < 30%.^12^ The mismatch ratio was determined as the ratio of the perfusion lesion volume to the volume of the ischemic core. Based on these thresholds, we then classified patients as “revised” target mismatch or no target mismatch as per the original volumetric criteria in the trial (absolute mismatch volume >10 ml, mismatch ratio >1.2, baseline ischemic core volume <70 ml).

### 2.2 Statistical analysis

Statistical analysis was performed with Stata version 15IC (StataCorp). Patients enrolled in the EXTEND trial were classified according to the target mismatch criteria. In patients who satisfied these criteria, we performed the analyses of clinical efficacy and safety outcomes as per the original EXTEND publication.^11^ In summary, dichotomous mRS and NIHSS‐based outcomes were analyzed using Poisson regression models with robust standard error estimation participant adjusted age and baseline NIHSS, with respective effect sizes reported as risk ratios (RRs). As per the original EXTEND paper, we also report the more traditional Odds Ratios (ORs) resulting from respective logistic regression models. Ordinal analysis of mRS was conducted across the full mRS scale with mRS 5 and 6 merged together, using ordinal logistic regression with the respective effect reported as common OR. *p*‐values < 0.05 were regarded as indicative of statistical significance. No correction for multiplicity of testing was made, consistent with the analysis in the main EXTEND paper.

## 3 RESULTS

From the 225 patients fulfilling the original RAPID mismatch criteria randomized in the EXTEND study, 29 (13%) failed to meet the autoMIStar revised target mismatch criteria. The reasons for failing to meet the revised mismatch criteria included: (i) a perfusion lesion (and hence penumbral volume) below <10 ml (*n* = 13); (ii) a large baseline ischemic core (>70 ml) which was calculated as <70 ml with RAPID (*n* = 9); and (iii) seven patients with an artifactual perfusion lesion volume by RAPID, but who had no perfusion lesion at all with autoMIStar (on expert review of raw data, the underlying causes were poor contrast injection *n* = 2, and motion artifact *n* = 3, and CT artifact = 2). Of the remaining 196 (87%) patients who fulfilled the revised target mismatch criteria, 103 received placebo and 93 alteplase. Of the 29 patients who did not fulfill revised target mismatch criteria, 11 received placebo and 18 received alteplase. Baseline characteristics of the original EXTEND cohort and the current revised target mismatch eligible patient populations are provided in Table 1.

**TABLE 1:** Original trial and current study patient demographics

	Original EXTEND target mismatch cohort	Revised target mismatch cohort	Patients not meeting revised target mismatch
Patient number	225 (113 alteplase, 112 placebo)	196 (93 alteplase, 103 placebo)	29 (18 alteplase, 11 placebo)
Age (median, IQR)	76 (64–81)	75 (64–81)	76 (64–79)
Baseline NIHSS (median, IQR)	11 (7–17)	12 (7–17)	8 (5–16)
24h NIHSS (median, IQR)	8 (4–15)	8 (4–15)	9.5 (2–16)
Automated CTP perfusion lesion volume (mL) (median, IQR)	68 (34–102)	42 (16–92)	17 (6–88)
Automated CTP ischemic core lesion volume (mL) (median, IQR)	8 (2.5–19)	9 (4–18)	1 (0–48)
24h infarct core volume (mL) (median, IQR)	23 (10–47)	23 (12–47)	5.5 (0–46)
Any PH	13 (5.7%)	9 (4.5%)	4 (12.5%)
sICH	8 (4%)	5 (2.5%)	3 (9%)

Note

Abbreviations: CTP, computed tomography perfusion; DDc, delay and dispersion corrected; IQR, interquartile range; NIHSS, National Institutes of Health Stroke Scale; PH, parenchymal hematoma; sICH, symptomatic intracranial hemorrhage.

Automated CTP perfusion lesion was measured by Tmax >6 s lesion with the original trial software algorithm, and was measured by Delay Time >3 s lesion with DDc algorithm.

**FIGURE 1:**
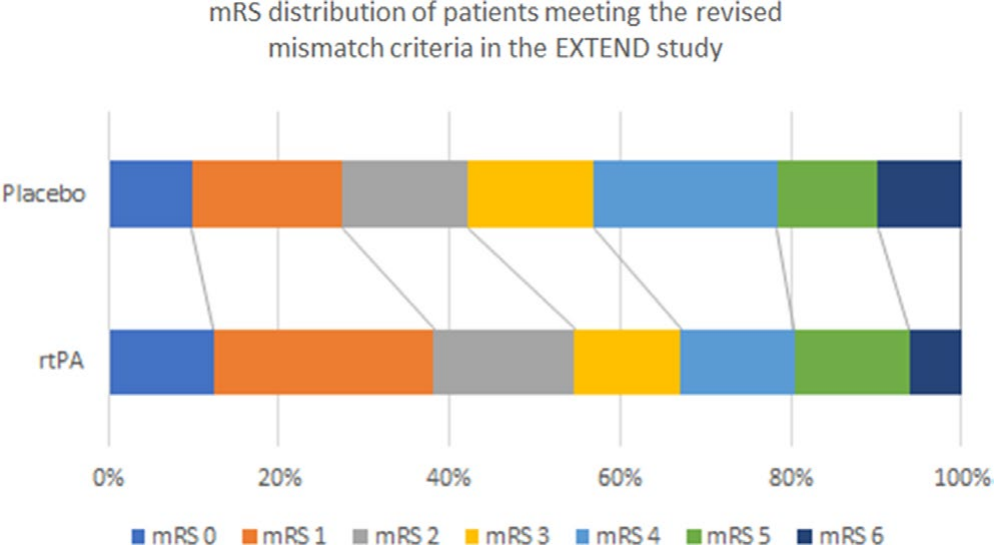
Modified Rankin Scale Scores at 90 days of the participants meeting the revised target mismatch criteria in the EXTEND study. Patients in the rtPA group have improved functional outcome at 90 days

Comparisons in outcomes between the original mismatch cohort and revised mismatch cohort are shown in Figure 1 and Table 2. Notably, in the revised target mismatch cohort there was a significant shift toward better outcomes across the whole mRS with alteplase compared to placebo (OR of ≥1 mRS category improvement = 1.87, CI 1.12, 3.11, *p* = 0.015). This was not significant in the original mismatch cohort (Figure 1). In the revised target mismatch cohort, 40% of the patients treated with alteplase achieving the excellent outcome (mRS 0–1) at 3‐month compared with 28% in patient treated with placebo. In contrast, in the original mismatch cohort, the rates of excellent outcome were 35% alteplase versus 30% placebo. The larger treatment effect from alteplase in patients who met revised target mismatch criteria also led to an absolute increase of 8% in excellent functional outcome compared to those treated with placebo (covariate‐adjusted OR = 2.23, CI 1.08–4.58, *p* = 0.028); which did not reach significance in the original target mismatch cohort (covariate‐adjusted OR = 1.88, CI 0.99–3.59, *p* = 0.056). However, with an alternative analysis (covariate‐adjusted modified Poisson regression), alteplase‐treated patients in the original mismatch cohort were more likely to reach mRS 0–1 at 3 months (adjusted Risk Ratio [aRR] = 1.44, CI 1.01–2.06, *p* = 0.042). This benefit was also convincingly seen in the revised mismatch cohort (aRR = 1.62, 95% CI 1.10–2.40, *p* = 0.014). From the 29 patients not meeting revised mismatch criteria, 40% of the 18 treated with alteplase had excellent functional outcome at 3 months versus 44% with placebo (aRR, 0.595, CI 0.08–4.22, *p* = 0.604). The revised perfusion lesion volumes were significantly smaller compared to the original RAPID volumes (median 68 ml IQR 34–102 ml vs. 42 ml 16–92 ml, *p* = 0.036). However, there was no significant difference in the ischemic core volume, likely due to these being relatively small volumes (Table 1).

**TABLE 2:** A comparison of the original EXTEND cohort with those who fulfilled the revised Target Mismatch criteria

Outcome	Original EXTEND Target (225)		Revised Target Mismatch cohort (196)	
	Placebo (112)	Alteplase (113)	Placebo (103)	Alteplase (93)
mRS 0 *n*, (%)	12 (10.7%)	14 (12.4%)	10 (9.7%)	12 (12.9%)
mRS 1 *n*, (%)	21 (18.8%)	26 (23.0%)	18 (17.5%)	25 (26.9%)
mRS 2 *n*, (%)	15 (13.4%)	16 (14.2%)	15 (14.6%)	16 17.2%)
mRS 3 *n*, (%)	16 (14.3%)	15 (13.3%)	15 (14.6%)	12 (12.9%)
mRS 4 *n*, (%)	24 (21.4%)	15 (13.3%)	22 (21.4%)	13 (14.0%)
mRS 5 *n*, (%)	14 (12.5%)	14 (12.4%)	12 (11.7%)	13 (14.0%)
mRS 6 *n*, (%)	10 (8.9%)	13 (11.5%)	10 (9.7%)	6 (6.5%)
mRS 0–1 Odds Ratio	1.88 (CI 0.99–3.59, *p* = 0.056)		2.23 (CI 1.08–4.58, *p* = 0.028)*	
mRS 0–1 Risk Ratio	1.44 (CI 1.01–2.06, *p* = 0.042)		1.62 (CI 1.10–2.40, *p* = 0.014)	
mRS 0–2 Odds Ratio	2.02 (CI 1.07–3.83, *p* = 0.031)		2.73 (CI 1.33–5.61, *p* = 0.006)	
mRS 0–2 Risk Ratio	1.36 (CI 1.06–1.76, *p* = 0.017)		1.54; (CI 1.18–2.01, *p* = 0.001)	
mRS 6 Odds Ratio	1.21 (CI 0.47– 3.12 *p*= 0.692)		0.64 (CI 0.21, 1.92, *p* = 0.434)	
mRS 6 Risk Ratio	1.17 (CI 0.57–2.4, *p* = 0.67)		0.73 (CI 0.30–1.78, *p* = 0.5)	
mRS shift (Odds ratio, reversed)	1.55 (CI 0.96–2.49, *p* = 0.382)		1.87 (CI 1.12–3.11, *p* = 0.015)*	
NIHSS change baseline to 24 hours	2.76 (CI 1.45–5.26, *p* = 0.006)		2.63 (CI 1.45–4.77, *p* < 0.001)	
Symptomatic intracranial hemorrhage OR	7.75 (CI 0.93, 64.95, *p* = 0.059)		4.19 (CI 0.53, 32.7, *p* = 0.171)	
Symptomatic intracranial hemorrhage RR	7.22 (CI 0.97, 53.54, *p* = 0.053)		4.43 (CI 0.50, 39.14, *p* = 0.181)	

Note

All odds ratios and relative risk scores and adjusted for baseline age and NIHSS as per the original EXTEND analyses.

In the original trial mismatch cohort, symptomatic intracranial hemorrhage (sICH) occurred in 7/113 (6%) of alteplase‐treated patients and 1/112 (1%) in the placebo group (aRR 7.22, 95% CI 0.97–53.54, *p* = 0.053). In the revised mismatch cohort, sICH occurred in 4/93 (4%) of alteplase‐treated patients and 1/103 (1%) in the placebo group (aRR 4.43 95% CI 0.50–39.14, *p* = 0.181). Thus, there were three more patients with sICH in the alteplase group from the original target mismatch cohort who did not fulfill revised target mismatch. Of these three patients, two had large baseline ischemic core volumes >70 ml with the revised autoMIStar analysis, and one had no target mismatch tissue, likely reflecting spontaneous reperfusion (before treatment) on subsequent expert review.

## 4 DISCUSSION

Fully automated perfusion software analysis with an alternative software revealed a proportion of patients who did not have target mismatch, and who also appeared less likely to benefit from thrombolysis in the EXTEND trial. Although the original EXTEND primary outcome of mRS 0–1 at 3 months (with covariate‐adjusted risk analysis) was met, the absolute benefit of alteplase in terms of excellent functional outcome compared to placebo was only 5%. Despite the reduction in sample size (29 less patients did not meet the revised mismatch criteria), alteplase‐treated patients who fulfilled the revised target mismatch criteria had a higher chance of better outcome compared to those seen in the entire EXTEND cohort, with an 8% absolute increase in excellent outcome. Nine (9/29) patients not meeting the revised mismatch criteria analysis had a large core (>70 ml) on re‐analysis. Two had fatal sICH (received alteplase), and none of the nine with core >70 ml after revised analysis achieved a good functional three‐month outcome. This is consistent with past studies showing this group have a poor natural history regardless of thrombolytic treatment.^13^ These results suggest that differences in automated perfusion software outputs are clinically relevant.

The finding of a considerable number of patients not fulfilling revised target mismatch criteria due to a large core by the autoMIStar Delay and Dispersion corrected algorithm, but which the RAPID software considered were mismatch eligible due to core <70 ml, is curious. Theoretically, failure to correct for Delay and Dispersion should lead to underestimation of CBF and hence overestimation of the ischemic core volume. This was certainly the case in a previous study where we tried to approximate the RAPID perfusion algorithm.^14^ However, clearly this is an oversimplification of what goes in the “black boxes” of proprietary software, and one needs to consider other differences between the software such as motion correction and post‐processing smoothing that generates the end‐product maps. Another factor might be the choice of CBF threshold for core, past validation work with RAPID suggests a CBF threshold of <38% might actually be the most accurate, and this would lead to larger core volume estimates.^15^

Thirteen patients that fulfilled original EXTEND trial mismatch criteria had total perfusion lesions (and hence penumbra) <10 ml after the revised analysis using DT (rather than Tmax) to estimate penumbra. The majority (11/13) of these patients had an excellent outcome, consistent with past data that suggests a good natural history in patients with small perfusion lesions (ie, without thrombolysis).^16^ However, two of these patients had a poor outcome with alteplase. On review of the perfusion maps, these patients had evidence of spontaneous reperfusion (hyperperfusion), but there was some residual adjacent hypoperfusion, as one sees if perfusion imaging is performed during the reperfusion process.^12^, ^15^ As previously described, it is possible such patients are at increased risk of hemorrhagic transformation with thrombolysis. Thus, it appears that a Delay and Dispersion corrected perfusion algorithm is less prone to overestimation of “penumbra” (although there may be other software differences as well that contribute to this overestimation of “penumbra”).

The alternative automated software also seemed to be less prone to produce artefactual perfusion lesions, with seven patients who were originally enrolled in EXTEND with target mismatch having no perfusion lesion after the alternative perfusion analysis. On expert review of the original EXTEND study automated perfusion‐core/penumbra maps the “perfusion lesion” was clearly artefactual, and was mostly related to slow/inadequate contrast injection, and/or motion artefact. This also highlights the pitfalls of purely relying on automated output from perfusion software (irrespective of vendor), and the need for stroke clinicians to carefully review the raw perfusion data.^17^ A related, further important clinical point apparent from this study is that clinicians must always carefully review the non‐contrast CT for acute ischemic changes (hypodensity), particularly in later time windows. If the patient is undergoing spontaneous reperfusion there may be infarct core that is visible on NCCT but not detected by perfusion imaging (as the core calculated by CTP requires the CBF to be low). The presence of acute ischemic change on NCCT might negate a “mismatch” on the automated perfusion software output. There was one such case in EXTEND that had acute hypodensity on NCCT but also had target mismatch according to RAPID. This patient did not fulfill target mismatch criteria after processing with the alternative software.

Although this is the first randomized trial using imaging‐based selection within which there has been a comparison of automated perfusion CT software, limitations include the relatively small trial sample size, and the post hoc nature of this analysis. However, the automated software analysis was performed blinded to all other data, including clinical outcomes and treatment allocation. Additionally, due to the post hoc nature of this analysis, we do not know if patients screened for the trial but excluded by lack of RAPID mismatch criteria (and hence not randomized), may have met the revised mismatch criteria and thus may have altered the results from this study. It is important to acknowledge that the findings of this study are specific to a particular post‐processing imaging technique (autoMIStar compared to RAPID), and as such, our results might not be direct translated to other perfusion software currently used.^18^, ^19^, ^20^

In conclusion, our results demonstrate that differences between automated perfusion software excludes some patients at the margins of target mismatch (both for small perfusion lesions and large core). This study heightens awareness of differences in automated perfusion imaging software outputs and emphasizes that the variability in automated perfusion software outputs does matter clinically. Nonetheless, the other key message of this analysis is that these data emphasize the positive results of the original EXTEND trial, and show a strong benefit for alteplase in the later time window/wake‐up stroke in patients with target mismatch.

## CONFLICT OF INTEREST

Dr. Davis, receiving advisory board fees from AstraZeneca and Boehringer Ingelheim; and Dr. Donnan, receiving advisory board fees from AstraZeneca Australia, Bayer, Boehringer Ingelheim, Merck, Pfizer, and Servier; Dr. Parsons reports receiving advisory board fees from Boehringer Ingelheim, and research partnerships with Canon Medical Systems, Apollo Medical Imaging, and Siemens. No other potential conflict of interest relevant to this article were reported.

## Data Availability

Anonymized data that support the findings of this study are available from the corresponding author upon reasonable request.
